# Phlegmonous Gastritis in a Patient With Nonalcoholic Steatohepatitis-Related Cirrhosis: A Case Report and Review of Literature

**DOI:** 10.7759/cureus.33551

**Published:** 2023-01-09

**Authors:** Norikazu Iwata, Yasushi Adachi, Yukinari Yoshida, Yoshifumi Ishii, Takao Endo

**Affiliations:** 1 Department of Internal Medicine and Gastroenterology, Sapporo Shirakabadai Hospital, Sapporo, JPN; 2 Department of Pathology, Sapporo Shirakabadai Hospital, Sapporo, JPN

**Keywords:** sepsis-induced liver dysfunction, moraxella (branhamella) catarrhalis, autopsy, nonalcoholic steatohepatitis-related cirrhosis, phlegmonous gastritis

## Abstract

It is sometimes difficult to diagnose phlegmonous gastritis clinically. We herein present a rare autopsy report of a patient with phlegmonous gastritis associated with nonalcoholic steatohepatitis-related cirrhosis. The patient died of hepatic failure two weeks after exacerbation of anorexia and rapid progression of liver dysfunction. Autopsy revealed cholangitis lenta and sepsis-induced liver dysfunction, which was attributed to phlegmonous gastritis due to *Moraxella (Branhamella) catarrhalis*. Phlegmonous gastritis has seldom been reported in patients with liver cirrhosis. We believe the importance of keeping in mind that phlegmonous gastritis could be one of the complications of advanced liver cirrhosis.

## Introduction

Phlegmonous gastritis is a nonspecific purulent inflammatory disease of the stomach that might involve only the submucosal layer or the full thickness of the stomach wall [1]. The risk factors for phlegmonous gastritis include alcohol abuse, diabetes mellitus, chronic gastritis, gastric ulcer, endoscopic treatments, and immunodeficient status [2]. The symptoms often include chills, fever, upper abdominal pain, nausea, and vomiting. However, the symptoms are sometimes mild, making diagnosis difficult.

Phlegmonous gastritis has been reported to be associated with various conditions, including gastric carcinoma/lymphoma, human immunodeficiency virus infection, Kaposi’s sarcoma, myeloma, leukemia, Sjogren’s syndrome, rheumatoid arthritis, hemodialysis, superior mesenteric artery syndrome, esophageal rupture, postsurgery, and patients treated with tumor necrosis factor (TNF) alpha receptor antagonists and chemotherapy [3-6]. Couveilhier provided the first autopsy report of phlegmonous gastritis in 1820 [7], and only one case of chronic hepatitis accompanied by phlegmonous gastritis has since been described [8]. Likewise, only one case report of liver cirrhosis (LC) with phlegmonous gastritis has been described [9]. We herein report a rare case of nonalcoholic steatohepatitis-related cirrhosis with sepsis-induced worsening of liver dysfunction secondary to phlegmonous gastritis.

## Case presentation

A 60-year-old male had been treated for nonalcoholic steatohepatitis-related cirrhosis at an outside hospital accredited by the Japan Society of Hepatology. He could not receive a hepatic transplant due to obesity (body weight: 111 kg; body mass index: 39 kg/m^2^). One month before admission to our hospital, he developed Miller-Fisher syndrome as a complicating condition. He was transferred to our hospital for the treatment of cirrhosis and rehabilitation. He remained on the waiting list for the hepatic transplant, and we were in contact with the transplant department. He had been treated for portal vein thrombosis and hepatocellular carcinoma (using radiofrequency ablation) six years before starting this treatment. Physical examination at his current presentation showed grade 1 hepatic encephalopathy, jaundice, and splenomegaly. Laboratory findings on admission showed pancytopenia (white blood cell count {WBC}: 2.0×10^9^/L; hemoglobin: 11.9 g/dL; platelets: 46×10^9^/L), hypoproteinemia (total protein: 6.9 g/dL; albumin: 1.8 g/dL), liver dysfunction (total bilirubin {T-Bil}: 4.5 mg/dL; aspartate aminotransferase {AST}: 53 U/L; alanine aminotransferase: 17 U/L), hyperammonemia (212 μg/dL), and low prothrombin time (29.5%) (Table 1).

**Table 1:** Laboratory data of the patient. This case was recognized as rapid progression of liver dysfunction in the last two weeks before death. WBC: white blood cell count; Hb: hemoglobin; Plt: platelets; TP: total protein; Alb: albumin; AST: aspartate aminotransferase; ALT: alanine aminotransferase; ɤ-GT: gamma-glutamyltransferase; T-Bil: total bilirubin; CRP: c-reactive protein; NH3: ammonia; PT: prothrombin time

Inspection item	Reference value		Unit	On admission	Day 125	Day 134	Day 141
	Lower limit	Upper limit					
WBC	3.5	9.7	x10^9^/L	2	1.5	1.72	4.82
Hb	13.6	18.3	g/dL	11.9	10.5	11.1	11.9
Plt	140	379	x10^9^/L	46	34	36	53
TP	6.5	8.2	g/dL	6.9	6.4	6.6	7
Alb	3.7	5.5	g/dL	1.8	1.6	1.6	1.6
AST	10	40	U/L	53	60	79	94
ALT	5	45	U/L	17	18	25	35
γ‐GT	0	79	U/L	10	9	16	20
T-Bil	0.3	1.2	mg/dL	4.5	4.4	8.4	22.8
CRP	0	0.3	mg/dL	0.3	0.3	0.19	3.48
NH_3_	30	86	μg/dL	212	262	175	154
PT	80	120	%	29.5	35.7	-	-

**Figure 1:**
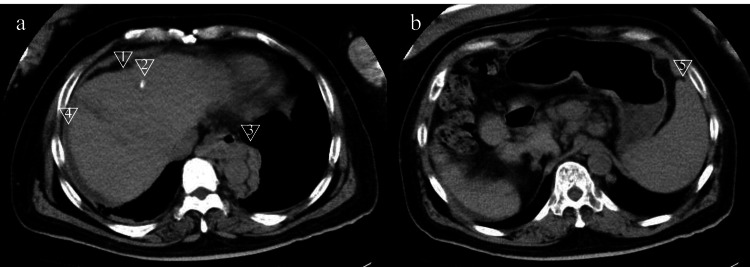
Computed tomography four months after admission. The images show (a) atrophic liver (arrow 1), a treated hepatocellular carcinoma nest (arrow 2), collateral veins (arrow 3), ascites (arrow 4); and (b) splenomegaly (arrow 5), which remained unchanged during hospitalization.

No antibodies against hepatitis B surface antigen, hepatitis B core antigen, or hepatitis C virus were detected. Child-Pugh class was C, Mac-2 binding protein glycosylation isomer was high (cutoff index: 17.95), and the indocyanine green retention rate at 15 min was 70.2%. Although protein concentration induced by vitamin K absence or antagonist II was high (786 mAU/mL), alpha-fetoprotein concentration was not (2.3 ng/dL). Both computed tomography (CT) and ultrasound revealed atrophic liver with a treated nodule, collateral vessels, ascites, and splenomegaly, but no gastric wall thickening (Figures 1a, 1b).

These findings did not change during this hospitalization. Esophagogastroduodenoscopy on hospital day 49 revealed chronic gastritis and grade A gastroesophageal reflux disease. The patient was treated with vonoprazan fumarate, ursodeoxycholic acid, levocarnitine chloride, branched-chain amino acid-enriched nutrients (Aminoleban EN; Otsuka Pharmaceutical Co., Tokyo, Japan), and spironolactone. No prominent changes in his clinical course were seen until clinical day 125. At this time, the patient experienced exacerbation of anorexia, but no fever, nausea, vomiting, epigastralgia, or diarrhea. His clouding of consciousness existed from the evening before death (The Glasgow Coma Scale {GCS} 9 points). His body temperature on the day of his death was 39.0°C. Sequential organ failure assessment (SOFA) score on the day of death was 11 points excluding one item (GCS=9, score 3; mean arterial pressure {MAP} 53 mmHg, score 1; PaO_2_/FiO_2_ not measured; platelets 53×10^9^/L, score 2; bilirubin 22.8 mg/dL, score 4; and creatinine 1.34 mg/dL, score 1). Thus he might be septic.

From day 125 to days 134 and 141, serum levels of T-Bil increased rapidly from 4.4 mg/dL to 8.4 and 22.8 mg/dL, aspartate aminotransferase (AST) increased from 60 U/L to 79 and 94 U/L, gamma-glutamyltransferase (γ-GT) increased from 9 U/L to 16 and 20 U/L, c-reactive protein (CRP) increased from 0.3 mg/dL to 0.19 and 3.48 mg/dL, and WBC increased from 1.50 ×10^9^/L to 1.72 and 4.82 ×10^9^/L. He died of progressive hepatic failure on day 141 (Table 1). Subsequently, autopsy was performed. Two major pathologic lesions were observed in the stomach and the liver.

**Figure 2:**
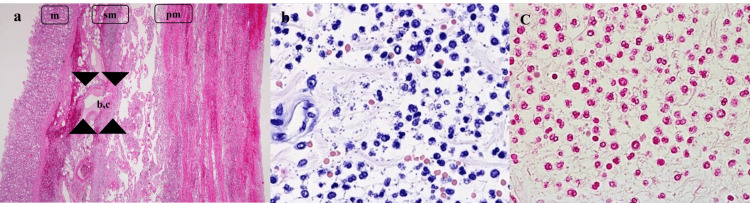
Microscopic view of the stomach. Endotoxin (+) Gram-negative diplococci (*Moraxella {Branhamella} catarrhalis*) had penetrated and eroded the stomach wall (arrow), (a) H&E stain, (b) Giemsa stain, and (c) Gram stain. (a) x40, (b, c) x400.

In the stomach, the mucosa of the lesion was swollen and hemorrhagic, and histologic examination showed diffuse phlegmonous gastritis, characterized by infiltration of the entire thickness of the gastric wall with neutrophils and Gram-negative diplococci. The bacteria were considered as probably being endotoxin-producing *Moraxella (Branhamella) catarrhalis* (Figures 2a-2c).

**Figure 3:**
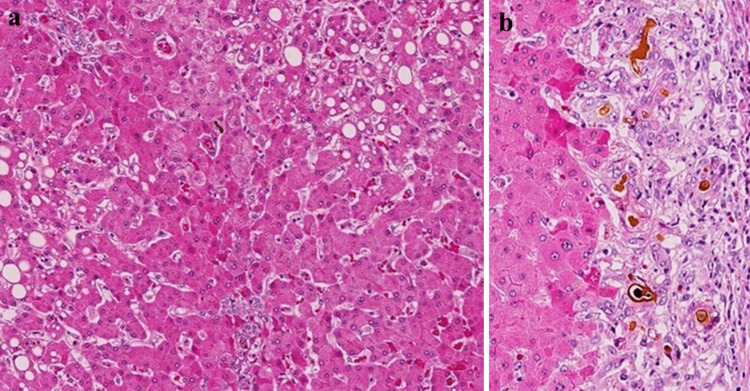
Microscopic view of the liver. The images show (a) nonalcoholic steatohepatitis-related liver cirrhosis (LC). Small nodular LC showing macrovesicular steatosis and injured, ballooned hepatic cells. (b) Histological analysis of cholangitis lenta showed proliferation and dilatation of the canals of Hering at the border of the portal vein, presence of bile thrombi, and infiltration of neutrophils and lymphocytes. Changes in the interlobular bile duct were scarce. (a) x40, (b) x200.

In the liver, micronodular cirrhosis corresponding to steatohepatitis-related cirrhosis was obvious, and small nodular liver cirrhosis and hepatic atrophy were observed. Macrovesicular steatosis and ballooned hepatocytes could be observed. Accordingly, this was indicative of nonalcoholic steatohepatitis-related cirrhosis and due to this additional occurrence of sepsis-induced liver dysfunction, the biliary pathways were considered likely to have been damaged by endotoxin, resulting in the degeneration and subsequent proliferation of dilated canals of Hering near the portal vein. However, few pathological changes were evident in the lobular bile ducts. Further, numerous bile thrombi were seen congesting and proliferating the canals of Hering, with scattered neutrophils and lymphocytes, corresponding to sepsis- or endotoxin-related cholangitis lenta (Figures 3a, 3b). This was the probable cause of death in this patient.

## Discussion

Phlegmonous gastritis can be caused by the following bacteria: Streptococcus*, Staphylococcus aureus, Escherichia coli, Haemophilus influenzae,* and Proteus species [10]. Phlegmonous gastritis caused by *B. catarrhalis* has never been previously reported. The mortality rate of phlegmonous gastritis is reportedly about 42% [11]. As *B. catarrhalis* is widely known to cause respiratory tract infection, a possible mode of infection, in this case, was direct gastric mucosal invasion after swallowing the pathogenic bacteria. Another possible reason for infection was lowered resistance due to malnutrition, since our patient with cirrhosis suffered from loss of appetite, leading to a compromise of the sterilizing ability of the stomach by acid secretion inhibitors. Moreover, this patient was immunocompromised due to decompensated cirrhosis [12].

In this case, cholangitis lenta and sepsis-induced liver dysfunction were present. Cholangitis lenta (also known as ductular cholestasis) is usually caused by Gram-negative bacteria [13]. The mechanisms by which cholestasis contributes to sepsis-induced liver dysfunction might include the production of inflammatory mediators and dysfunctional bile excretion caused by endotoxin translocation [12,14]. Another mechanism might be lipopolysaccharide-impaired organic anion transportation inside the capillary bile ducts [12,15]. In addition, cholangitis lenta caused by inflammatory cells invading around capillary bile ducts [13] and sepsis-associated intrahepatic cholestasis with bile thrombi in capillary bile ducts have been reported [16].

On the other hand, the possibilities of translocated routes other than phlegmonous gastritis might exist. For example, this patient had cholangitis, which could be a bacterial translocation route. However, acute cholangitis by *B. catarrhalis* has never been reported previously. In histological examination, numerous bile thrombi, neutrophils, and lymphocytes were found to proliferate the canals of Hering, indicating the sepsis-related cholangitis lenta. Moreover, *B. catarrhalis *existed with neutrophils in all layers of the stomach. Thus, it was most likely that phlegmonous gastritis was orally developed, which caused sepsis and then cholangitis.

Table 2 summarizes two cases in the literature of phlegmonous gastritis associated with chronic hepatic disease in addition to the present case [8,9]. The underlying hepatic diseases were alcoholic liver disease and LC of unknown etiology. Both those patients were less than 60 years of age and had chronic liver disease that progressed rapidly and had a fatal course. However, each case was caused by a different bacterium.

**Table 2:** Previous cases of phlegmonous gastritis associated with chronic hepatic disease. Those patients were less than 60 years of age and had chronic liver disease that progressed rapidly and had a fatal course. However, each case was caused by a different bacterium. UN: unknown; LC: liver cirrhosis; NASH-LC: nonalcoholic steatohepatitis-related cirrhosis

Case no.	Age	Sex	Liver disease	Symptom	Sepsis	Treatments	Pathological findings	Bacteria	Outcome	Reference no.
1	UN	UN	Alcoholic liver disease	Ascites	(-)	Antibiotics	Peritonitis	Haemophilus infuluenzae	Autopsy	[8]
2	50	Male	LC of unknown causes	Abdominal pain, vomiting, tarry stool	(+)	UN	Liver lobules surrounded by fibrous tissue showing lymphocytic infiltration	Hemolytic streptococcus	Autopsy	[9]
3	60	Male	NASH-LC	Anorexia	(+)	none	Small nodular LC and cholangitis lenta	G (-) diplococci *Moraxella (Branhamella) catarrhalis*	Autopsy	This case

Hence, phlegmonous gastritis should be recognized as a severe complication of chronic liver disease. Elevated T-Bil levels were previously reported as an indicator of poor prognosis in sepsis-induced liver dysfunction [17]. Additionally, γ-GT levels serve as an early marker of prognosis. Although our patient did not have fever or inflammatory findings, both T-Bil and γ-GT levels increased in the last two weeks before death. The reason why did both bilirubin and γ-GT levels increase might be that cholestasis persisted in the parenchyma and bile infarction occurred.

The following diagnostic modalities are reportedly useful for diagnosing phlegmonous gastritis: ultrasound, esophagogastroduodenoscopy, and ultrasonic endoscopy [18]. In our case, if the patient had undergone esophagogastroduodenoscopy at an earlier stage of phlegmonous gastritis, the outcome might have been different. Thus, we believe that the accumulation and analysis of more cases are important for elucidating liver dysfunction secondary to phlegmonous gastritis.

## Conclusions

We have reported a rare autopsy case of nonalcoholic steatohepatitis-related cirrhosis that caused sepsis-induced liver dysfunction via phlegmonous gastritis. Phlegmonous gastritis should be recognized as an important complication of chronic liver disease, given the possible lack of specific symptoms and capacity for rapid progression.
